# Space Use and Movements During Egg Laying Associated With Nest Fate and Female Survival in Eastern Wild Turkeys

**DOI:** 10.1002/ece3.73026

**Published:** 2026-02-02

**Authors:** Paige E. Goodman, Nicholas W. Bakner, Nickolas A. Gulotta, Erin E. Ulrey, Bret A. Collier, Michael J. Chamberlain

**Affiliations:** ^1^ Warnell School of Forestry and Natural Resources University of Georgia Athens Georgia USA; ^2^ College of Agriculture and Natural Resources University of Delaware Newark Delaware USA; ^3^ School of Renewable Natural Resources Louisiana State University Agricultural Center Baton Rouge Louisiana USA

**Keywords:** egg laying, *Meleagris gallopavo*, nesting, reproduction, space use, survival, wild Turkey

## Abstract

Reproduction is a fundamental aspect of a species' life history that is energetically costly, yet critical for population sustainability and genetic diversity. Wild turkeys exhibit high rates of nest loss and female mortality during reproduction, prompting females to make decisions related to spatial and movement decisions during nesting. Using GPS data from eastern wild turkeys ( *Meleagris gallopavo silvestris* ), we assessed female movements and space use during laying and evaluated potential impacts of those metrics on nest success and female survival during incubation. We used a Bayesian logistic regression to estimate nest success and female survival based on space use, daily movements, and range overlap with conspecifics during the laying period. We found that with each increase of ~700 m in average daily distance traveled during laying, there was a 1.73% decrease in the probability of nest success. We also found that having a greater number of conspecific females with overlapping core areas had a positive influence on nest fate. Specifically, an increase of 1 overlapping female (one standard deviation) was associated with a 4.76% increase in the probability of nest success. Conversely, we found weak support that female survival was positively related to increasing average daily distances traveled. Our findings suggest that female wild turkeys perceive reproductive advantages to sharing space with conspecifics during the laying period. Conversely, our findings suggest that movements of female wild turkeys within their reproductive period may only weakly influence metrics of reproductive success during both laying and incubation.

## Graphical abstract

We assessed behaviors of female wild turkeys during the laying period and evaluated impacts of those behaviors on nest success and female survival. We found that increased movements during laying resulted in a decreased probability of nest success but an increased probability of female survival during incubation.

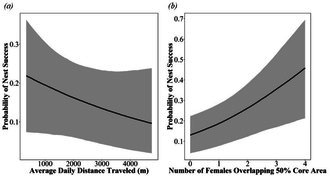

## 1 Introduction

Reproduction is a fundamental aspect of a species' life history that is energetically costly, yet critical for population sustainability and genetic diversity (Avise 1996). Resource allocation theory governs how females allocate resources between producing offspring and individual growth and maintenance. Resource allocation posits that resources allocated to one aspect of an organism's life, such as survival, cannot simultaneously be allocated to another, such as reproduction. Consequently, females must balance energetic demands of producing offspring with resource acquisition and evading predation. Among avian taxa, the mortality rates of females during the nesting period play a significant role in the choice of reproductive tactics (Fontaine and Martin 2006), leading to a gradient of reproductive tactics where individuals prioritize productivity and survival differently (Jones 1989). The initiation of egg laying and incubation represents critical phases for avian species, demanding spatial confinement and considerable energy expenditure (Deeming and Reynolds 2015). For uniparental incubators, females must balance remaining at the nest site and making recess movements away from the nest location to secure resources (Williams 1996).

During incubation, uniparental ground nesters exhibit behaviors similar to those of central place foragers (Orians and Pearson 1979). When individuals are tethered to a centralized location like a nest, there may be space use restrictions and availability of resources may be limited (Kacelnik 1984; Van Gils and Tijsen 2007; Lalla et al. 2022). In species with relatively long incubation periods, individuals may be confined for extended temporal periods, requiring them to prioritize resource acquisition and minimize predation risk. To minimize predation risk during incubation, ground nesters often choose nest locations in dense vegetation or use behavioral tactics to avoid or hide from predators (Wiebe and Martin 1997; Smith et al. 2012). Individuals may familiarize themselves with certain areas to identify the less risky choices to lay their nest, and balance finding places with foraging resources while avoiding drawing attention from predators (Olsson et al. 2008). Likewise, individuals may also be drawn to similar areas on the landscape due to shared preferences for particular vegetation types. However, individuals must be aware of proximity to conspecifics, as spatial association can facilitate the acquisition or exchange of information about predators, resources, or social cues (Samplonius et al. 2017; Shen et al. 2017), which ultimately may prompt them to adopt various space use and movement tactics during incubation (Doligez et al. 2002; Hatchwell 2009). However, being near conspecifics may expose the individual to increased predation risk and conspecific parasitism, which has been documented in multiple species of ground nesting birds (Yom‐Tov 2001; Krakauer 2008; Deeming and Reynolds 2015; Sullivan et al. 2022).

Female eastern wild turkeys ( *Meleagris gallopavo silvestris* , hereafter wild turkey) are uniparental ground‐nesters that have long incubation periods (e.g., 25–30 days) during which they must identify suitable foraging opportunities and concealment spots to evade predators effectively (Healy 1992; Conley et al. 2015). Ground‐nesting coupled with extended incubation periods, along with a reliance on crypsis for survival and exposure to multiple predator guilds, results in increased vulnerability to nest loss by females (Blomberg et al. 2015). Throughout incubation, female wild turkeys exhibit central place foraging behavior, making periodic foraging excursions from the nest site (Conley et al. 2015; Bakner et al. 2019, 2024; Lohr et al. 2020). To survive the incubation period and reproduce successfully, female wild turkeys must identify resource‐rich areas that offer both foraging opportunities and predator concealment (Green 1982; Martin 1998).

A suite of studies has examined how vegetation structure at and around nest sites affects nest success; however, the extant literature remains inconclusive, with reported results being contradictory (Byrne and Chamberlain 2013; Yeldell et al. 2017; Bakner et al. 2019; Lohr et al. 2020; Keever et al. 2022). Similarly, early studies inferred that female wild turkeys prospected for nest locations (Badyaev 1995; Chamberlain and Leopold 2000), but contemporary studies have shown that such prospecting does not occur (Conley et al. 2015; Collier et al. 2019). However, although prenesting and laying ranges may overlap little, female wild turkeys display increased daily movements and reduced space use during the laying phase (Schofield 2019). Such movement patterns are consistent with prospecting behavior, whereby individuals explore local conditions to identify suitable nest sites and profitable foraging patches prior to incubation (Pärt and Doligez 2003). Under movement‐based decision frameworks, behavioral decisions are expected to reflect information gained through prior space use rather than assumptions about site familiarity per se (Matthiopoulos et al. 2005). Specifically, during laying but before entering the incubation phase, female wild turkeys engage in prospecting behavior, exploring their surroundings to assess resource distribution (Bakner et al. 2024), which may facilitate efficient travel to and from resources while minimizing mortality risks (Piper 2011; Wakefield et al. 2015).

The nesting ecology of female wild turkeys has been extensively studied. Contemporary literature has noted that turkey populations exhibit high rates of nest loss (Byrne and Chamberlain 2013; Crawford et al. 2021), prompting females to adopt spatial and movement decisions to combat these issues (Lohr et al. 2020). However, despite extensive research on nest locations and vegetation at nest sites (Porter 1992; Fuller et al. 2013; Streich et al. 2015; Little et al. 2016), the behaviors of female wild turkeys during the laying period remain relatively unstudied (Bakner et al. 2024). For instance, it is plausible that movements exhibited by female wild turkeys during the laying period could impact nest success and female survival, both of which can influence population trajectories (Vangilder and Kurzejeski 1995; Lehman et al. 2022). Bakner et al. (2024) demonstrated that the number of times females revisited areas within their incubation ranges did not influence nest fate, but rather, the number of patches visited during incubation recesses did. However, it is unclear if female movements during laying influence metrics of nest success and female survival during incubation.

Our objectives were to describe space use of female eastern wild turkeys during the egg laying period, including home range and core area sizes, average daily distance traveled, and overlap in space use with other females, and to relate these metrics to nest success and female survival during incubation. Contemporary studies continue to demonstrate that movements of wild turkeys can have important fitness consequences related to both individual survival and reproductive success (Lohr et al. 2020; Gulotta et al. 2024, 2025a, 2025b). Therefore, we hypothesized that metrics of space use and movements by females during the laying period would influence nest success and female survival rates during incubation. Specifically, we predicted that females who traveled greater daily distances and had larger home ranges and core areas during laying would experience greater individual survival and rates of nest success, as increased movement may enhance their knowledge of local resources and escape routes, thereby reducing their risk of predation. Secondly, we hypothesized that maintaining core areas that overlapped with other females would affect nest success and female survival rates during incubation. We predicted that females who maintained overlapping core areas during the laying period would have decreased individual survival and nest success rates because of increased attraction of predators to the area.

## 2 Methods

### 2.1 Study Area

We conducted research across study sites in Georgia, South Carolina, and Louisiana, USA. In Georgia, we conducted research on 3 wildlife management areas (WMAs), including Silver Lake, Cedar Creek, and B.F. Grant WMAs. The Silver Lake WMA, located in southwest Georgia, was owned and managed by the Georgia Department of Natural Resources—Wildlife Resources Division (GADNR). Silver Lake WMA was dominated by mature pine (*Pinus* spp.) forests and forested wetlands. Overstory species were predominately longleaf pine ( *P. palustris* ), loblolly pine ( *P. taeda* ), slash pine ( *P. echinata* ), oaks (*Quercus* spp.), and sweetgum ( *Liquidambar styraciflua* ). Prescribed fire was applied on an approximately 2‐ to 3‐year return interval. Cedar Creek WMA was owned by the US Forest Service (USFS) and managed in partnership with the Georgia Department of Natural Resources‐Wildlife Resources Division (GADNR). Cedar Creek was composed primarily of loblolly pine uplands, mixed hardwood‐pine forests, and hardwood lowlands containing mostly oaks, sweetgum, and hickory (*Carya* spp.). Prescribed fire was applied to pine‐dominated stands on an approximately 3–5‐year return interval. B.F. Grant was owned by the Warnell School of Forestry and Natural Resources at the University of Georgia and managed jointly by GADNR and the Warnell School. B. F. Grant was dominated by loblolly pine stands, agricultural lands, mixed hardwood‐pine forest, and hardwood lowlands of similar species composition as Cedar Creek.

Our two study sites in South Carolina included the Webb WMA Complex and the Savannah River Site (SRS). We conducted research on the Webb WMA Complex from January 2014–March 2018. The Webb WMA Complex was a combination of three adjacent WMAs (Webb, Palachacola, and Hamilton Ridge). These WMAs covered a 10,483‐ha area and were owned and managed by the South Carolina Department of Natural Resources (SCDNR) and consisted primarily of longleaf, loblolly, and slash pine forests with hardwood stands along riparian corridors, and expanses of bottomland hardwood wetlands. Management activities included prescribed fire, timber management, fallow field management, and maintaining agricultural field plots focused on enhancing habitat for wildlife species. During 2021–2023, we conducted research on SRS, which was a 78,000‐ha tract owned by the United States Department of Energy and jointly managed by the USFS. Approximately 94% of SRS was dominated by loblolly, longleaf, and bottomland hardwood forests. The remaining forested areas included forested swamps, riparian areas, and mixed hardwood stands. Approximately 30% of SRS was managed for red cockaded woodpeckers ( *Picoides borealis* ), with prescribed fire applied on a 3–5 year return interval. For detailed descriptions of site conditions on SRS and Webb WMA see Wightman et al. (2019).

We also conducted research on two sites in Louisiana. The western Louisiana (WLA) site included Kisatchie National Forest (KNF) and Fort Johnson Wildlife Management Area (FJWMA). The KNF was owned and managed by the United States Forest Service (USFS), whereas FJWMA was jointly owned by the USFS and the United States Army. Both sites were composed of forest dominated by pines, hardwood bottoms, and forested wetlands, and included forest openings and roads throughout the sites. Our study site in southeastern Louisiana (SELA) included a broad collection of private properties and Sandy Hollow WMA owned by the Louisiana Department of Wildlife and Fisheries. This study site consisted of rolling hills with longleaf pine plantations, hardwood riparian zones, and agricultural areas used primarily for grazing and row crops.

### 2.2 Data Collection

We captured female wild turkeys using rocket nets baited with cracked corn during January–March from 2014 to 2023. We classified captured individuals as juvenile or adult based on barring on the ninth and tenth primary feathers (Dickson 1992). We marked each individual with an aluminum rivet tarsal band (National Band and Tag company, Newport, Kentucky, USA) and a backpack style GPS/UHF transmitter (e‐obs GmbH Grünwald, Germany) or GPS/VHF transmitter (Lotek UK Ltd., Wareham, Dorset, UK). All individuals were released at the capture location immediately following processing. The capture, handling, and marking procedures were approved by the Institutional Animal Care and Use Committee at the University of Georgia (Protocol Numbers A2014 06‐008‐R2, A2014 06‐008‐R2, A2019 01‐025‐R2, A2020 06‐018‐R1, and A2021 11‐024‐Y1‐A0) and Louisiana State University (Protocol Numbers A2014‐013, A2015‐07, and A2018‐13).

We programmed Lotek transmitters to record 1 GPS location nightly to provide a roost location and hourly GPS locations from 0500 to 2000 (Standard Time and according to the appropriate time zones) for the duration of the study (Cohen et al. 2018). We programmed e‐obs transmitters to record hourly GPS locations from 0400 to 2000 for the duration of the study and used the 0400 point as the roost location. Units collected data until the battery died or the individual died, and the transmitter was retrieved. To monitor the status and locations of all birds, we used radiotelemetry via a UHF handheld command unit or VHF receiver. We used a BaseStation (e‐obs GmbH Grünwald, Germany) or a PinPoint VHF Commander (Lotek UK Ltd., Wareham, Dorset, UK) to remotely download data from transmitters at least once a week from January to July. During the nesting period, we increased the monitoring and data downloads of females to ≥ 2 times per week. We viewed GPS locations and determined incubation initiation by noting when female location became centralized around a single point (Conley et al. 2015; Yeldell et al. 2017; Wood et al. 2019). We did not disturb the nests while females were incubating. Instead, we continued to monitor them daily from a distance > 20 m via VHF/UHF. Upon nest termination, we located the nest site to determine if hatching had occurred and considered nests successful if ≥ 1 egg hatched (Conley et al. 2016). We continued to monitor females for additional nest attempts until reproductive activity ceased.

### 2.3 Analysis

We performed data processing and analysis in program R (v.4.3.1; R Development Core Team 2023). We processed and cleaned the raw GPS data by removing locations that had dilution of precision values (DOP) > 7. To determine dates of the onset of the egg laying and incubation period, we mapped our spatial–temporal data using FireTail (Schäuffelhut Berger GmbH, Unterhaching, Germany) and ArcPro (Environment Systems Research Institute, Redlands, California, USA). We determined locations for each nesting attempt by when an individual's locations became concentrated around a single point for several days (Guthrie et al. 2011; Conley et al. 2015; Yeldell et al. 2017; Wood et al. 2019). We determined the initiation of incubation as the first time an individual remained on the nest overnight (Bakner et al. 2019; Lohr et al. 2020) and then evaluated hourly locations for the previous 20 days to determine when a female initially visited the nest site. After identifying an incubating bird, we used a 27.5 m buffer (accounting for conservative GPS error; Bakner et al. 2019) to identify the date of first visit. We considered this first visit to the nest as the date of nest initiation and the beginning of the laying period. We recognize that this methodology could be biased by a day if the female visited the nest prior, but we based this approach on previously published works (Conley et al. 2016; Collier et al. 2019).

### 2.4 Covariates

For each female, we estimated average daily distance traveled for the laying period for each nesting attempt by summing the total distance traveled daily within the laying period (number of days laying) divided by length (in days) of the laying period. We calculated 95% home ranges and 50% core areas during the laying period by fitting dynamic Brownian bridge movement models (dBBMMs) to the time‐specific location data (Cohen et al. 2018) using package move (Kranstauber et al. 2018) in program R (v.4.3.1; R Core Team 2023). We used an error estimate of 20 m, a moving window size of 7 locations, and a margin setting of 3 locations (Byrne et al. 2014; Cohen et al. 2018). We kept window and margin size constant to account for changes in GPS sampling frequency because we failed to see any measurable effects of altering these values when we began our analysis (Cohen et al. 2018).

**FIGURE 1:**
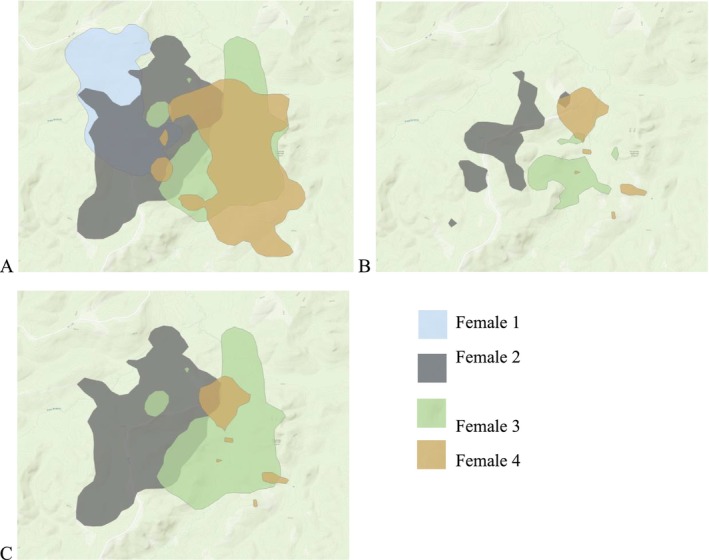
95% and 50% utilization ranges during the laying period for four female eastern wild turkeys to depict how covariates used to evaluate overlap in space use among females were determined. (A) depicts overlapping 95% utilization (home ranges), (B) depicts overlapping 50% utilization (core areas), and (C) depicts 95% home ranges that overlap to 50% core area.

We quantified range overlap by categorizing whether simultaneously monitored females had overlapping ranges (intersected = 1, no intersection = 0). Specifically, we calculated these metrics across three different combinations: 95% home range that overlapped with another 95% home range, 50% core area that overlapped with another 50% core area, and 50% core area that overlapped with a 95% home range (Figure 1). Additionally, we calculated how many individual females overlapped with one another during the laying period using these same three combinations (Figure 1).

In preliminary analyses, we assessed the correlations among all covariates using Pearson's correlation coefficient. We found that average daily distance traveled, home range size, and core area size were highly correlated (*r* = 0.79–0.86), suggesting significant multicollinearity among these spatial metrics. Similarly, substantial correlations were observed among the overlap variables (*r* = 0.67–0.92). To mitigate effects of multicollinearity and simplify the model, we selected average daily distance traveled to represent the spatial movement metrics and the number of females overlapping core areas to represent the overlap measures in our final analyses. This selection was based on their relevance to our research objectives and their comprehensive representation of the respective variable groups.

### 2.5 Nest Fate and Female Survival Models

We constructed two separate Bayesian logistic regression models to test our hypothesis regarding the effect of female movements and space use during the laying period on nest fate and female survival during incubation. For both models we included fixed effects of average daily distance traveled, total number of individual females who overlapped core areas during the laying period, nesting attempt, and a random effect of individual wild turkey. We treated the probability of nest fate and survival (success or failure; alive or dead) as a Bernoulli distribution. To aid model fit and allow for direct comparison of effect sizes of each predictor variable, we standardized all fixed effects included in the models using the scale function in R (v.4.3.1; R Core Team 2023). We fitted models using package brms in program R (Bürkner 2017). We computed 4 MCMC chains for 8000 iterations, discarding the first 1000 iterations as a burn‐in (Gelman and Rubin 1992). We calculated 95% credible intervals (hereafter CrI) that provided a metric of uncertainty and present the median of the posterior distribution. We then computed the probability of direction which describes the proportion of the posterior distribution that is in the direction of the estimated effect (Makowski, Ben‐Shachar, Chen, et al. 2019; Makowski, Ben‐Shachar, and Lüdecke 2019). We interpret effects where the CrI does not overlap zero or with a probability of direction ≥ 89% as showing support for an effect (Makowski, Ben‐Shachar, Chen, et al. 2019, Makowski, Ben‐Shachar, and Lüdecke 2019). Additionally, for statistically significant effects with effect estimates near zero, we calculated the region of practical equivalence (ROPE), which estimates the proportion of the posterior distribution that lies within a range considered too small to have biological relevance (Makowski, Ben‐Shachar, Chen, et al. 2019, Makowski, Ben‐Shachar, and Lüdecke 2019). For both models, we inspected trace and density plots, and all estimated parameters had R‐hat values < 1.1, indicating that all chains converged (Gelman 2004).

## 3 Results

We used 872 nesting attempts (initial attempts = 626, renesting attempts = 246) by 643 females (567 adults, 76 juveniles) for our nest fate and female survival analysis. Of the 872 nests, 210 (24%) hatched and 662 (76%) failed. Of the 643 females that initiated nests, 82 (13%) were depredated during incubation. Home range size during the laying period ranged from 3.02 ha to 584.11 ha with a mean of 16.39 ha, and core area size ranged from 0.62 ha to 73.31 ha with a mean of 16.39 ha (Table 1). Average daily distance traveled during the laying period ranged from 334.06 m to 4761.46 m with a mean of 2151.14 m (Table 1).

**TABLE 1:** Mean average daily distance traveled (m), 95% home range, 50% core area (ha), number of females who overlapped 50% core areas with core area of a female, number of females who overlapped 95% home ranges with the home range of a female, and number of females who overlapped 95% home ranges with the core area of a female with associated standard deviations (SD) for female eastern wild turkeys during the egg laying period across the southeastern United States during 2014–2023.

Covariate	Mean	SD
Average daily distance traveled (m)^a^	2151.14	699.93
50% Core area (ha)^b^	16.39	10.09
95% Home range (ha)^c^	89.67	59.32
Mean number of females that overlapped core areas^d^	0.47	0.78
Mean number of females that overlapped home ranges^e^	0.75	1.06
Mean number of females with home range that overlapped core area^f^	0.60	0.91

^a^ Average daily distance traveled by the hen during the laying period.

^b^ 50% daily utilization during laying period determined by dBBMM.

^c^ 95% daily utilization during laying period determined by dBBMM.

^d^ Number of females whose 50% utilization overlap during laying period.

^e^ Number of females whose 95% utilization overlap during laying period.

^f^ Number of females whose 95% utilization overlaps the 50% core area of the female.

**FIGURE 2:**
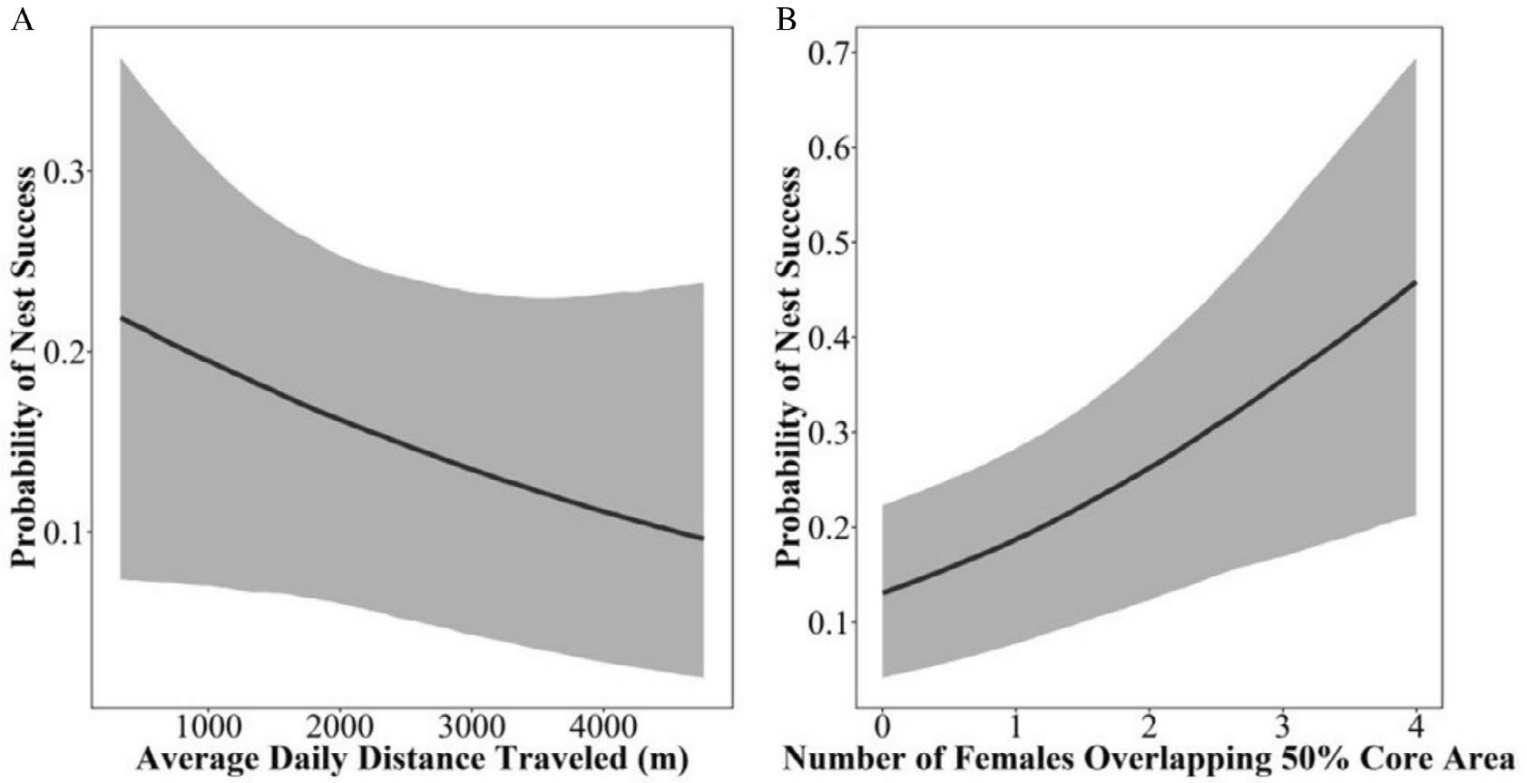
Probability of nest success for female wild turkeys as a function of (A) average daily distance traveled (m) and (B) the number of females who had overlapping 50% core areas with a female during the laying period. Error bars and gray areas represent the 95% credible intervals.

For nest fate, we found support for an increase in average daily distance traveled negatively influencing nest fate (β = −0.15, 95% CrI = −0.43–0.09, PD = 89.47%; Table 2, Figure 2). Although most of the posterior distribution for daily distance traveled during laying fell within the ROPE (60.09%), the effect cannot be conclusively considered biologically negligible. Specifically, with each increase of approximately 700 m (one standard deviation of variable, Table 1) in average daily distance traveled during laying, there was a 1.73% decrease in the probability of nest success, a minimal magnitude of effect that may not reflect a biologically meaningful relationship. We also found support that the total number of females that overlapped core areas (β = 0.34, 95% CrI = 0.11–0.65, PD = 99.84%; Table 2, Figure 2) positively influenced nest fate. An increase of 1 overlapping female (one standard deviation) was associated with a 4.76% increase in the probability of nest success. Lastly, we found no evidence that nest fate varied across nesting attempts (Table 2).

**TABLE 2:** Posterior means, 95% credible intervals, and probability of direction (pd) statistic for covariates used to model nest fate for female eastern wild turkeys across the southeastern United States during 2014–2023. Estimates > 0 positively influenced daily nest survival, whereas estimates < 0 negatively influenced survival.

Covariate	Lower 95% CI	Estimate	Upper 95% CI	pd
Intercept	−2.81	−1.69	−1.11	100
Average daily distance traveled^a^	−0.43	−0.15	0.09	89.47
Number of females 50% overlapping^b^	0.11	0.34	0.65	99.84
Nesting Attempt				
Nest Attempt 2	−0.30	0.36	1.40	83.40
Nest Attempt 3	−1.11	0.33	2.18	65.67

^a^ Average daily distance traveled by the female during the laying period.

^b^ Number of females whose 50% utilization overlap during laying period.

**FIGURE 3:**
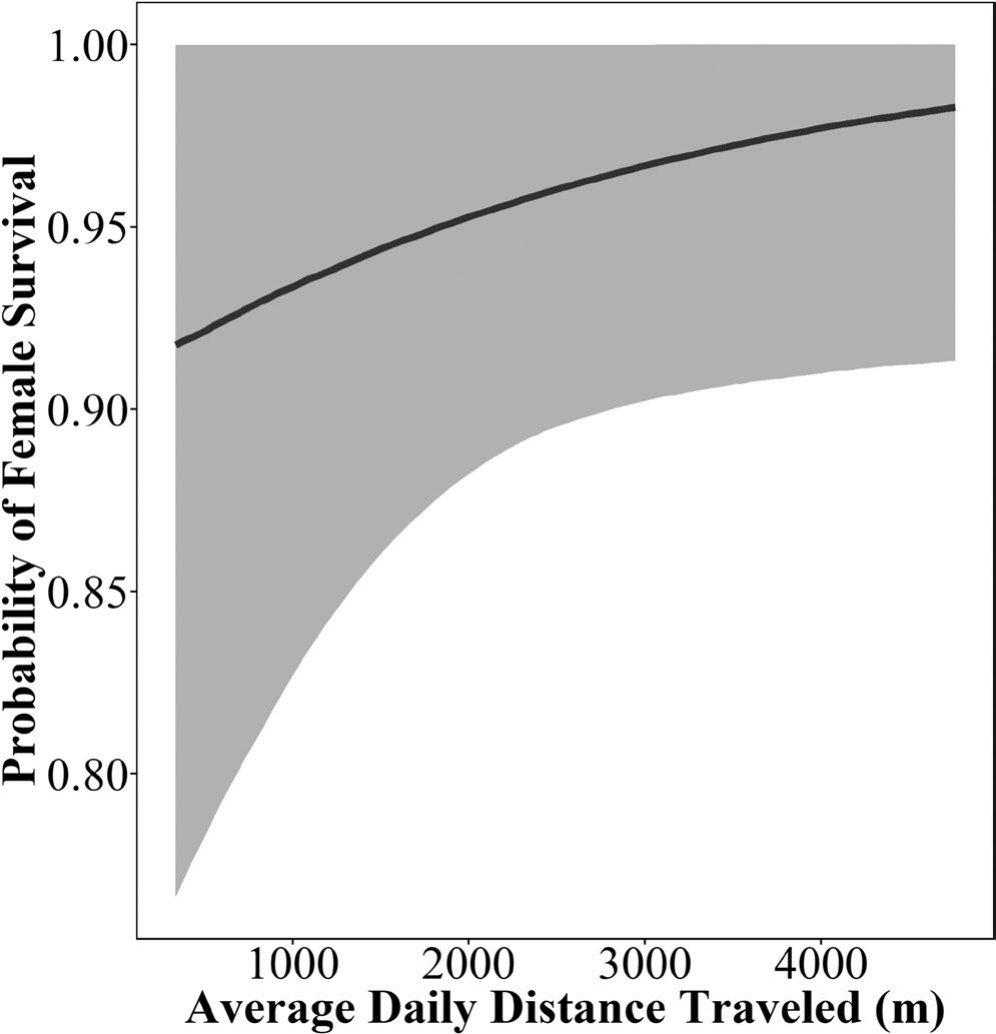
Probability of survival for female wild turkeys during incubation as a function of the average daily distance traveled (m) during the laying period. Gray areas represent the 95% credible intervals.

For female survival during incubation, we found weak support that an increase in average daily distance traveled during laying positively influenced female survival (β = 0.25, 95% CrI = −0.13–0.70, PD = 92.39%, ROPE = 33.30%; Table 3, Figure 3). Specifically, with each increase of approximately 700 m (one standard deviation of variable, Table 1) traveled daily there was a 0.93% increase in the probability of female survival during incubation, which is likely not a biologically meaningful effect. We observed no effects of the number of females that overlapped 50% core areas during the laying period on female survival while incubating (Table 3). Likewise, we found no differences in female survival across nesting attempts (Table 3).

**TABLE 3:** Posterior means, 95% credible intervals, and probability of direction (pd) statistic for covariates measured during the laying period and used to model survival of female wild turkeys during incubation across the southeastern United States during 2014–2023. Estimates > 0 positively influenced survival, whereas estimates < 0 negatively influenced survival.

Covariate	Lower 95% CI	Estimate	Upper 95% CI	pd
Intercept	2.06	3.06	9.82	100.00
Average daily distance traveled^a^	−0.13	0.25	0.70	92.39
Number of females 50% overlapping^b^	−0.16	0.17	0.75	84.33
Nesting Attempt				
Nest Attempt 2	−1.68	0.38	1.24	73.90
Nest Attempt 3	−4.23	−0.37	1.22	66.84

^a^ Average daily distance traveled by the female during the laying period.

^b^ Number of females whose 50% utilization overlap during laying period.

## 4 Discussion

We used hourly spatial locations to describe space use and movements of female eastern wild turkeys during the egg laying period and subsequently related those spatial behaviors to individual survival and nest success during the incubation period. We found that daily distances traveled by females during laying only weakly influenced both nest fate and female survival during incubation. We also found that overlapping core areas with conspecifics during egg laying positively influenced nest fate. Our findings contribute to a growing body of literature suggesting that movements and space use of female wild turkeys during portions of their reproductive period may influence metrics of reproductive success (Fontaine and Martin 2006), and specific to wild turkeys, such tactics are occurring during both egg laying and incubation (Bakner et al. 2019; Lohr et al. 2020).

The egg laying period is significant as it represents a temporal period when female movements are restricted, therefore limiting their space use and access to available resources (Kacelnik 1984; Van Gils and Tijsen 2007). We found that on average female wild turkeys traveled 2151 m per day during the egg laying period and maintained home ranges and core area sizes of 90 ha and 16 ha, respectively. We note that only a single previous study evaluated movements of female turkeys during laying, with that work reporting larger range sizes which included the eastern and Rio Grande wild turkey subspecies (Schofield 2019). However, Schofield (2019) used a 99% utilization distribution to calculate home range sizes, whereas we used a 95% utilization distribution to calculate home range size as is consistent with other studies using dBBMM (Byrne et al. 2014; Cohen et al. 2018). Previous studies on spring home range sizes in other Galliformes have shown that species inhabiting open landscapes, such as sharp‐tailed grouse ( *Tympanuchus phasianellus* ), sage grouse ( *Centrocercus urophasianus* ), and black grouse ( *Lyrurus tetrix* ), often have home ranges > 150 ha (Ballard and Robel 1974; Rippin and Boag 1974; Wallestad and Pyrah 1974; Svedarsky 1979), whereas those in denser, forested habitats, like ruffed grouse ( *Bonasa umbellus* ), spruce grouse ( *Canachites canadensis* ), western capercaillie ( *Tetrao urogallus* ), and blue grouse ( *Dendragapus obscurus* ), maintain home ranges < 50 ha (Herzog and Boag 1978; Maxson 1978; Hannon et al. 1982; Wegge 1985; Wegge and Rolstad 1986). Therefore, the home ranges we observed for female wild turkeys during egg laying are comparable to what has been reported in other species using a similar mating system during the breeding and reproductive periods.

Movements during reproductive periods can influence individual metrics of fitness and reproductive success (Lyon and Anderson 2003; Beck et al. 2006). Females must balance the energetic demands of producing offspring, resource acquisition, and evading predators, which leads to spatial and movement decisions (Jones 1989; Boggs 1992; Fontaine and Martin 2006; Deeming and Reynolds 2015). Our findings showed weak support for a negative association between an increase in average daily distance traveled by females during egg laying and nest fate, as well as a weak positive influence on female survival. Although these effect sizes were small, the observed patterns were similar to findings by Lohr et al. (2020), who demonstrated females who took longer incubation recesses during the incubation period had lower nest success but greater individual survival. Increased daily movement may reflect exploratory behaviors related to resource assessment or predator avoidance; however, any such effects appear limited in magnitude and should be interpreted cautiously (Williams 1996; Gehr 2020; Bakner et al. 2024). Alternatively, some nesting sites could be in areas that are more resource‐rich than others, requiring females that use resource‐rich nesting areas to travel lesser distances than their counterparts. As such, we cannot rule out that resources were heterogeneously distributed across the landscape, and resource distribution could be driving the observed pattern of greater nest loss with increasing distances traveled.

The presence of conspecifics can influence female behaviors and reproductive success in a variety of avian species (Ramsey et al. 1999; Kivelä et al. 2014). Previous research demonstrates that female birds may use conspecifics as cues to select nesting sites, and that social information from conspecifics positively influences reproductive success (Forsman et al. 2007; Hoi and Griggio 2012). We found that increases in the number of overlapping core areas with conspecific females positively influenced nest success in wild turkeys, which suggests that females perceive reproductive advantages to sharing space with conspecifics during the laying period. We speculate that these findings may stem from females benefiting from information acquisition relative to predation risk and resource distribution, which has been demonstrated in various bird species (Thornton and Raihani 2008; Farine et al. 2012; Firth and Sheldon 2016; Samplonius et al. 2017). However, increased overlap may also arise from nonsocial processes, such as multiple females independently selecting similar nesting cover or landscape features that confer higher nest success (Doligez et al. 2002; Danchin et al. 2004), or from predator swamping effects that reduce per‐capita predation risk in areas of higher nest density (Lima 2009). Because we did not document direct interactions among females and could not account for unmarked conspecifics, we caution that the spatial overlap we observed should not be interpreted as evidence of coordinated or cooperative behavior. Future research explicitly quantifying social interactions, habitat features, and predator responses will be necessary to disentangle these mechanisms.

We reached two primary conclusions from our work, including that increased movements by females only weakly influence nest success and female survival rates during incubation, and that overlapping space use with conspecifics can positively influence reproduction in wild turkeys. Positive fitness consequences via interactions with conspecifics have been observed in other avian species (Ramsey et al. 1999; Kivelä et al. 2014; Joly et al. 2024) likely reflecting a combination of social and nonsocial processes operating across heterogeneous landscapes, rather than a single mechanism driving movement and spatial overlap (Doligez et al. 2002; Danchin et al. 2004; Lima 2009). Furthermore, various factors can influence conspecific interactions in wild turkeys, including social dominance and relatedness. Previous research noted that female turkeys join social groups prior to breeding composed mostly of unrelated individuals (Watkins et al. 2025), and contemporary research suggests that these social groups tend to nest in the same area (Ulrey et al. 2023). Future research should explicitly identify the social, behavioral, and habitat‐driven mechanisms that link movement behavior and spatial overlap to variation in vital rates, particularly nest success and female survival, during the reproductive period.

## Author Contributions

**Paige E. Goodman:** conceptualization (equal), data curation (lead), formal analysis (equal), investigation (supporting), methodology (supporting), writing – original draft (lead). **Nicholas W. Bakner:** conceptualization (supporting), formal analysis (supporting), methodology (supporting), writing – review and editing (supporting). **Nickolas A. Gulotta:** formal analysis (supporting), methodology (supporting), writing – review and editing (supporting). **Erin E. Ulrey:** formal analysis (supporting), methodology (supporting), writing – review and editing (supporting). **Bret A. Collier:** formal analysis (supporting), methodology (supporting), writing – review and editing (supporting). **Michael J. Chamberlain:** conceptualization (supporting), funding acquisition (lead), investigation (supporting), project administration (lead), supervision (lead), writing – review and editing (supporting).

## Funding

This work was supported by the Georgia Department Of Natural Resources and South Carolina Department of Natural Resources.

## Ethics Statement

This research was conducted with approval from the Institutional Animal Care and Use Committee at the University of Georgia (Protocol #A2014 06‐008‐R2, A2014 06‐008‐R2, A2019 01–025‐R2, A2020 06‐018‐R1, and A2021 11‐024‐Y1‐A0) and Louisiana State University (Protocol #A2014‐013, A2015‐07, and A2018‐13).

## Conflicts of Interest

The authors declare no conflicts of interest.

## Data Availability

Data and code used here are available on Dryad https://doi.org/10.5061/dryad.9p8cz8wz3.

## References

[ece373026-bib-0001] Avise, J. C. 1996. “Three Fundamental Contributions of Molecular Genetics to Avian Ecology and Evolution.” Ibis 138: 16–25.

[ece373026-bib-0002] Badyaev, A. V. 1995. “Nesting Habitat and Nesting Success of Eastern Wild Turkeys in the Arkansas Ozark Highlands.” Condor 97: 221–232.

[ece373026-bib-0003] Bakner, N. W. , L. R. Schofield , C. Cedotal , M. J. Chamberlain , and B. A. Collier . 2019. “Incubation Recess Behaviors Influence Nest Survival of Wild Turkeys.” Ecology and Evolution 9: 14053–14065. 31938503 10.1002/ece3.5843 PMC6953688

[ece373026-bib-0004] Bakner, N. W. , E. E. Ulrey , B. A. Collier , and M. J. Chamberlain . 2024. “Prospecting During Egg Laying Informs Incubation Recess Movements of Eastern Wild Turkeys.” Movement Ecology 12: 4. 38229127 10.1186/s40462-024-00451-3 PMC10792941

[ece373026-bib-0005] Ballard, W. P. , and R. J. Robel . 1974. “Reproductive Importance of Dominant Male Greater Prairie Chickens.” Auk 91: 75–86.

[ece373026-bib-0006] Beck, J. L. , K. P. Reese , J. W. Connelly , and M. B. Lucia . 2006. “Movements and Survival of Juvenile Greater Sage Grouse in Southeastern Idaho.” Wildlife Society Bulletin 34: 1070–1078.

[ece373026-bib-0007] Blomberg, E. J. , D. Gibson , and J. S. Sedinger . 2015. “Biases in Nest Survival Associated With Choice of Exposure Period: A Case Study in North American Upland Game Birds.” Condor 117: 577–588.

[ece373026-bib-0008] Boggs, C. L. 1992. “Resource Allocation: Exploring Connections Between Foraging and Life History.” Functional Ecology 6: 508–518.

[ece373026-bib-0009] Bürkner, P.‐C. 2017. “Brms: An R Package for Bayesian Multilevel Models Using Stan.” Journal of Statistical Software 80: 1–28.

[ece373026-bib-0010] Byrne, M. E. , and M. J. Chamberlain . 2013. “Nesting Ecology of Wild Turkeys in a Bottomland Hardwood Forest.” American Midland Naturalist 170: 95–110.

[ece373026-bib-0011] Byrne, M. E. , J. C. McCoy , J. W. Hinton , M. J. Chamberlain , and B. A. Collier . 2014. “Using Dynamic Brownian Bridge Movement Modelling to Measure Temporal Patterns of Habitat Selection.” Journal of Animal Ecology 83: 1234–1243. 24460723 10.1111/1365-2656.12205

[ece373026-bib-0012] Chamberlain, M. J. , and B. D. Leopold . 2000. “Habitat Sampling and Selection by Female Wild Turkeys During Preincubation.” Wilson Bulletin 112: 326–331.

[ece373026-bib-0013] Cohen, B. S. , T. J. Prebyl , B. A. Collier , and M. J. Chamberlain . 2018. “Home Range Estimator Method and GPS Sampling Schedule Affect Habitat Selection Inferences for Wild Turkeys.” Wildlife Society Bulletin 42: 150–159.

[ece373026-bib-0014] Collier, B. A. , N. Fyffe , A. Smallwood , et al. 2019. “Reproductive Ecology of Gould's Wild Turkeys (*Meleagris gallopavo Mexicana*) in Arizona.” Wilson Journal Of Ornithology 131: 667–679.

[ece373026-bib-0015] Conley, M. , J. Oetgen , J. Barrow , M. Chamberlain , K. Skow , and B. Collier . 2015. “Habitat Selection, Incubation, and Incubation Recess Ranges of Nesting Female Rio Grande Wild Turkeys in Texas.” National Wild Turkey Symposium 11: 117–126.

[ece373026-bib-0016] Conley, M. D. , N. A. Yeldell , M. J. Chamberlain , and B. A. Collier . 2016. “Do Movement Behaviors Identify Reproductive Habitat Sampling for Wild Turkeys?” Ecology and Evolution 6: 7103–7112. 28725385 10.1002/ece3.2401 PMC5513226

[ece373026-bib-0017] Crawford, J. C. , W. F. Porter , M. J. Chamberlain , and B. A. Collier . 2021. “Wild Turkey Nest Success in Pine‐Dominated Forests of the Southeastern United States.” Journal of Wildlife Management 85: 498–507.

[ece373026-bib-0018] Danchin, É. , L. A. Giraldeau , T. J. Valone , and R. H. Wagner . 2004. “Public Information: From Nosy Neighbors to Cultural Evolution.” Science 305: 487–491. 15273386 10.1126/science.1098254

[ece373026-bib-0019] Deeming, D. C. , and S. J. Reynolds . 2015. Nests, Eggs, and Incubation: New Ideas About Avian Reproduction. Oxford University Press.

[ece373026-bib-0020] Dickson, J. G. 1992. “Introduction.” In The Wild Turkey: Biology and Management, edited by J. G. Dickson . Stackpole Books.

[ece373026-bib-0021] Doligez, B. , E. Danchin , and J. Clobert . 2002. “Public Information and Breeding Habitat Selection in a Wild Bird Population.” Science 297: 1168–1170. 12183627 10.1126/science.1072838

[ece373026-bib-0022] Farine, D. R. , C. J. Garroway , and B. C. Sheldon . 2012. “Social Network Analysis of Mixed‐Species Flocks: Exploring the Structure and Evolution of Interspecific Social Behaviour.” Animal Behaviour 84: 1271–1277.

[ece373026-bib-0023] Firth, J. A. , and B. C. Sheldon . 2016. “Social Carry‐Over Effects Underpin Trans‐Seasonally Linked Structure in a Wild Bird Population.” Ecology Letters 19: 1324–1332. 27623746 10.1111/ele.12669 PMC5082527

[ece373026-bib-0024] Fontaine, J. J. , and T. E. Martin . 2006. “Habitat Selection Responses of Parents to Offspring Predation Risk: An Experimental Test.” American Naturalist 168: 811–818. 10.1086/508297 17109323

[ece373026-bib-0025] Forsman, J. T. , R. L. Thomson , and J. T. Seppänen . 2007. “Mechanisms and Fitness Effects of Interspecific Information Use Between Migrant and Resident Birds.” Behavioral Ecology 18: 888–894.

[ece373026-bib-0026] Fuller, A. K. , S. M. Spohr , D. J. Harrison , and F. A. Servello . 2013. “Nest Survival of Wild Turkeys *Meleagris Gallopavo Silvestris* in a Mixed‐Use Landscape: Influences at Nest Site and Patch Scales.” Wildlife Biology 19: 138–146.

[ece373026-bib-0027] Gehr, B. 2020. “Stay Home, Stay Safe—Site Familiarity Reduces Predation Risk in a Large Herbivore in Two Contrasting Study Sites.” Journal of Animal Ecology 89: 1329–1339. 32144759 10.1111/1365-2656.13202

[ece373026-bib-0028] Gelman, A. 2004. “Parameterization and Bayesian Modeling.” Journal of the American Statistical Association 99: 537–545.

[ece373026-bib-0029] Gelman, A. , and D. B. Rubin . 1992. “Inference From Iterative Simulations Using Multiple Sequences.” Statistical Science 7: 457–511.

[ece373026-bib-0030] Green, H. E. 1982. “Reproductive Behavior of Female Wild Turkeys in Northern Lower Michigan.” Journal of Wildlife Management 46: 1065–1071.

[ece373026-bib-0031] Gulotta, N. A. , P. H. Wightman , B. A. Collier , and M. J. Chamberlain . 2024. “The Role of Human Hunters and Natural Predators in Shaping the Selection of Behavioural Types in Male Wild Turkeys.” Royal Society Open Science 11: 240788. 39508001 10.1098/rsos.240788 PMC11539261

[ece373026-bib-0032] Gulotta, N. A. , P. H. Wightman , B. A. Collier , and M. J. Chamberlain . 2025a. “Harvest and Natural Predation Shape Selection for Behavioural Predictability in Male Wild Turkeys.” Journal of Animal Ecology 94: 2627–2640. 41090990 10.1111/1365-2656.70157 PMC12673237

[ece373026-bib-0033] Gulotta, N. A. , P. H. Wightman , B. A. Collier , and M. J. Chamberlain . 2025b. “Testing the Human Shield Hypothesis: Female Wild Turkeys Have Greater Fitness Near Human Activity.” Journal of Applied Ecology 00: e70235.

[ece373026-bib-0034] Guthrie, J. D. , M. E. Byrne , J. B. Hardin , et al. 2011. “Evaluation of a Global Positioning System Backpack Transmitter for Wild Turkey Research.” Journal of Wildlife Management 75: 539–547.

[ece373026-bib-0035] Hannon, S. J. , L. G. Sopuck , and F. C. Zwickel . 1982. “Spring Movements of Female Blue Grouse: Evidence for Socially Induced Delayed Breeding in Yearlings.” Auk 99: 687–694.

[ece373026-bib-0036] Hatchwell, B. J. 2009. “The Evolution of Cooperative Breeding in Birds: Kinship, Dispersal and Life History.” Philosophical Transactions of the Royal Society, B: Biological Sciences 364: 3217–3227. 10.1098/rstb.2009.0109 PMC2781872 19805429

[ece373026-bib-0100] Healy, W. M. 1992. “Behavior.” In The Wild Turkey: Biology and Management, edited by J. G. Dickson , 46–64. Stackpole Books.

[ece373026-bib-0037] Herzog, P. W. , and D. A. Boag . 1978. “Dispersion and Mobility in a Local Population of Spruce Grouse.” Journal of Wildlife Management 42: 853–865.

[ece373026-bib-0038] Hoi, H. , and M. Griggio . 2012. “Bearded Reedlings Adjust Their Pair‐Bond Behaviour in Relation to the Sex and Attractiveness of Unpaired Conspecifics.” PLoSONE 7: e32806. 10.1371/journal.pone.0032806 PMC3290599 22393449

[ece373026-bib-0039] Joly, S. , A. McKellar , S. Mahoney , et al. 2024. “The Influence of Conspecific and Heterospecific Neighbours on Avian Reproductive Success.” Behaviour 161: 399–416.

[ece373026-bib-0040] Jones, G. 1989. “Optimizing Time Off the Nest During Incubation in Female Swallows (*Hirundo rustica* [L.]).” Functional Ecology 3: 303–309.

[ece373026-bib-0041] Kacelnik, A. 1984. “Central Place Foraging in Starlings ( *Sturnus vulgaris* ). I. Patch Residence Time.” Journal of Animal Ecology 53: 283–299.

[ece373026-bib-0042] Keever, A. C. , B. A. Collier , M. J. Chamberlain , and B. S. Cohen . 2022. “Early Nest Initiation and Vegetation Density Enhance Nest Survival in Wild Turkeys.” Ornithology 140: 1–14.

[ece373026-bib-0043] Kivelä, S. M. , J. Seppänen , O. Ovaskainen , et al. 2014. “The Past and the Present in Decision‐Making: The Use of Conspecific and Heterospecific Cues in Nest Site Selection.” Ecology 95: 3428–3439.

[ece373026-bib-0044] Krakauer, A. H. 2008. “Sexual Selection and the Genetic Mating System of Wild Turkeys.” Condor 110: 1–12.

[ece373026-bib-0045] Kranstauber, B. , M. Smolla , and A. K. Scharf . 2018. move: Visualizing and analyzing animal track data. R package version 3.1.0.

[ece373026-bib-0046] Lalla, K. M. , K. C. Fraser , B. Frei , et al. 2022. “Central‐Place Foraging Poses Variable Constraints Year‐Round in a Neotropical Migrant.” Movement Ecology 10: 1–12. 36127732 10.1186/s40462-022-00337-2 PMC9487155

[ece373026-bib-0047] Lehman, C. P. , M. J. Yarnell , A. R. Litt , C. T. Rota , and J. J. Rotella . 2022. “Factors Influencing Rate of Decline in a Merriam's Wild Turkey Population.” Journal of Wildlife Management 86: e22240.

[ece373026-bib-0048] Lima, S. L. 2009. “Predators and the Breeding Bird: Behavioral and Reproductive Flexibility Under the Risk of Predation.” Biological Reviews 84: 485–513. 19659887 10.1111/j.1469-185X.2009.00085.x

[ece373026-bib-0049] Little, A. R. , N. P. Nibbelink , M. J. Chamberlain , L. M. Conner , and R. J. Warren . 2016. “Eastern Wild Turkey Nest Site Selection in Two Frequently Burned Pine Savannas.” Ecological Processes 5: 1–10.

[ece373026-bib-0050] Lohr, A. K. , J. A. Martin , G. T. Wann , B. S. Cohen , B. A. Collier , and M. J. Chamberlain . 2020. “Behavioral Strategies During Incubation Influence Nest and Female Survival of Wild Turkeys.” Ecology and Evolution 10: 11752–11765. 33144998 10.1002/ece3.6812 PMC7593161

[ece373026-bib-0051] Lyon, A. G. , and S. H. Anderson . 2003. “Potential Gas Development Impacts on Sage Grouse Nest Initiation and Movement.” Wildlife Society Bulletin 31: 486–491.

[ece373026-bib-0052] Makowski, D. , M. S. Ben‐Shachar , S. A. Chen , and D. Lüdecke . 2019. “Indices of Effect Existence and Significance in the Bayesian Framework.” Frontiers in Psychology 10: 2767. 31920819 10.3389/fpsyg.2019.02767 PMC6914840

[ece373026-bib-0053] Makowski, D. , M. S. Ben‐Shachar , and D. Lüdecke . 2019. “bayestestR: Describing Effects and Their Uncertainty, Existence and Significance Within the Bayesian Framework.” Journal of Open Source Software 4: 1541.

[ece373026-bib-0054] Martin, T. E. 1998. “Are Microhabitat Preferences of Coexisting Species Under Selection and Adaptive?” Ecology 79: 656–670.

[ece373026-bib-0055] Matthiopoulos, J. , J. Harwood , and L. E. N. Thomas . 2005. “Metapopulation Consequences of Site Fidelity for Colonially Breeding Mammals and Birds.” Journal of Animal Ecology 74: 716–727.

[ece373026-bib-0056] Maxson, S. J. 1978. “Spring Home Range and Habitat Use by Female Ruffed Grouse.” Journal of Wildlife Management 42: 6–71.

[ece373026-bib-0057] Olsson, O. , J. S. Brown , and K. L. Helf . 2008. “A Guide to Central Place Effects in Foraging.” Theoretical Population Biology 74: 22–33. 18550139 10.1016/j.tpb.2008.04.005

[ece373026-bib-0058] Orians, G. H. , and N. E. Pearson . 1979. “On the Theory of Central Place Foraging.” Analysis of Ecological Systems 2: 157–177.

[ece373026-bib-0059] Pärt, T. , and B. Doligez . 2003. “Gathering Public Information for Habitat Selection: Prospecting Birds Cue on Parental Activity.” Proceedings of the Royal Society of London. Series B: Biological Sciences 270: 1809–1813. 10.1098/rspb.2003.2419 PMC1691451 12964983

[ece373026-bib-0060] Piper, W. H. 2011. “Making Habitat Selection More “Familiar”: A Review.” Behavioral Ecology and Sociobiology 65: 1329–1351.

[ece373026-bib-0061] Porter, W. F. 1992. “Habitat requirements.” In The Wild Turkey: Biology and Management, edited by J. G. Dickson , 202–213. Stackpole Books.

[ece373026-bib-0062] R Development Core Team . 2023. R: A Language and Environment for Statistical Computing. R Foundation for Statistical Computing.

[ece373026-bib-0063] Ramsey, S. M. , K. Otter , and L. G. Ratcliffe . 1999. “Nest‐Site Selection by Female Black‐Capped Chickadees: Settlement Based on Conspecific Attraction?” Auk 116: 604–617.

[ece373026-bib-0064] Rippin, A. B. , and D. A. Boag . 1974. “Recruitment to Populations of Male Sharp‐Tailed Grouse.” Journal of Wildlife Management 38: 616–621.

[ece373026-bib-0065] Samplonius, J. M. , I. M. Kromhout Van Der Meer , and C. Both . 2017. “Nest Site Preference Depends on the Relative Density of Conspecifics and Heterospecifics in Wild Birds.” Frontiers in Zoology 14: 56. 29270207 10.1186/s12983-017-0246-5 PMC5738223

[ece373026-bib-0066] Schofield, L. R. 2019. Evaluation of reproductive phenology and ecology of wild turkey (*Meleagris gallopavo*) across the southeastern United States. Thesis, Louisiana State University, Baton Rouge, USA.

[ece373026-bib-0067] Shen, S. F. , S. T. Emlen , W. D. Koenig , and D. R. Rubenstein . 2017. “The Ecology of Cooperative Breeding Behaviour.” Ecology Letters 20: 08–720. 10.1111/ele.12774 28480586

[ece373026-bib-0068] Smith, P. A. , I. Tulp , H. Schekkerman , H. G. Gilchrist , and M. R. Forbes . 2012. “Shorebird Incubation Behaviour and Its Influence on the Risk of Nest Predation.” Animal Behaviour 84: 835–842.

[ece373026-bib-0069] Streich, M. M. , A. R. Little , M. J. Chamberlain , L. M. Conner , and R. J. Warren . 2015. “Habitat Characteristics of Eastern Wild Turkey Nest and Ground‐Roost Sites in 2 Longleaf Pine Forests.” Journal of the Southeastern Association of Fish and Wildlife Agencies 2: 164–170.

[ece373026-bib-0070] Sullivan, D. J. , P. H. Wightman , B. A. Collier , and M. J. Chamberlain . 2022. “Instances of Intraspecific Nest Parasitism in Eastern and Rio Grande Wild Turkeys.” Wildlife Society Bulletin 46: e1276.

[ece373026-bib-0071] Svedarsky, W. D. 1979. Spring and summer ecology of female greater prairie chickens in northwestern Minnesota. Dissertation, University of North Dakota, Grand Forks.

[ece373026-bib-0072] Thornton, A. , and N. J. Raihani . 2008. “The Evolution of Teaching.” Animal Behaviour 75: 1823–1836.

[ece373026-bib-0073] Ulrey, E. E. , M. J. Chamberlain , and B. A. Collier . 2023. “Reproductive Asynchrony Within Social Groups of Female Eastern Wild Turkeys.” Ecology and Evolution 13: e10171. 37325717 10.1002/ece3.10171 PMC10266966

[ece373026-bib-0074] Van Gils, J. , and W. Tijsen . 2007. “Short‐Term Foraging Costs and Long‐Term Fueling Rates in Central‐Place Foraging Swans Revealed by Giving‐Up Exploitation Times.” American Naturalist 169: 609–620. 10.1086/513114 17427132

[ece373026-bib-0075] Vangilder, L. D. , and E. W. Kurzejeski . 1995. “Population Ecology of the Eastern Wild Turkey in Northern Missouri.” Wildlife Monographs 130: 1–48.

[ece373026-bib-0076] Wakefield, E. D. , I. R. Cleasby , S. Bearhop , et al. 2015. “Long‐Term Individual Foraging Site Fidelity—Why Some Gannets Don't Change Their Spots.” Ecology 96: 3058–3074. 27070024 10.1890/14-1300.1

[ece373026-bib-0077] Wallestad, R. , and D. Pyrah . 1974. “Movement and Nesting of Sage Grouse Hens in Central Montana.” Journal of Wildlife Management 38: 630–633.

[ece373026-bib-0078] Watkins, S. A. , B. M. VonHoldt , B. A. Collier , and M. J. Chamberlain . 2025. “Role of Kinship in Sociality of Female Eastern Wild Turkeys.” Wildlife Society Bulletin 49: e1630.

[ece373026-bib-0079] Wegge, P. 1985. “Spacing Pattern and Habitat Use of Capercaillie Hens in Spring.” Proceedings of the International Grouse Symposium 3: 261–277.

[ece373026-bib-0080] Wegge, P. , and J. Rolstad . 1986. “Size and Spacing of Capercaillie Leks in Relation to Social Behavior and Habitat.” Behavioral Ecology and Sociobiology 19: 401–408.

[ece373026-bib-0081] Wiebe, K. L. , and K. Martin . 1997. “Effects of Predation, Body Condition and Temperature on Incubation Rhythms of White‐Tailed Ptarmigan *Lagopus leucurus* .” Wildlife Biology 3: 219–227.

[ece373026-bib-0082] Wightman, P. H. , J. C. Kilgo , M. Vukovich , et al. 2019. “Gobbling Chronology of Eastern Wild Turkeys in South Carolina.” Journal of Wildlife Management 83: 325–333.

[ece373026-bib-0083] Williams, J. B. 1996. “Energetics of Avian Incubation.” In Avian Energetics and Nutritional Ecology, edited by C. Carey . Springer.

[ece373026-bib-0084] Wood, J. D. , B. S. Cohen , L. M. Conner , B. A. Collier , and M. J. Chamberlain . 2019. “Nest and Brood Site Selection of Eastern Wild Turkeys.” Forest Ecology and Management 411: 203–212.

[ece373026-bib-0085] Yeldell, N. A. , B. S. Cohen , A. R. Little , B. A. Collier , and M. J. Chamberlain . 2017. “Nest Site Selection and Nest Survival of Eastern Wild Turkeys in a Pyric Landscape.” Journal of Wildlife Management 81: 1073–1083.

[ece373026-bib-0086] Yom‐Tov, Y. 2001. “An Updated List and Some Comments on the Occurrence of Intraspecific Nest Parasitism in Birds.” Ibis 143: 133–143.

